# Nuclear receptor CAR-ERα signaling regulates the estrogen sulfotransferase gene in the liver

**DOI:** 10.1038/s41598-020-61767-9

**Published:** 2020-03-19

**Authors:** MyeongJin Yi, Muluneh Fashe, Shingo Arakawa, Rick Moore, Tatsuya Sueyoshi, Masahiko Negishi

**Affiliations:** Pharmacogenetics Section, Reproductive and Developmental Biology Laboratory, National Institute of Environmental Health Sciences, National Institutes of Health, Research Triangle Park, North Carolina, 27709 USA

**Keywords:** Cell biology, Molecular biology

## Abstract

Estrogen sulfotransferase (SULT1E1) inactivates estrogen and regulates its metabolic homeostats. Whereas SULT1E1 is expressed low in the liver of adult mice, it is induced by phenobarbital (PB) treatment or spontaneously in diabetic livers *via* nuclear receptors. Utilizing constitutive active/androstane receptor (CAR) KO, estrogen receptor α (ERα KO, phosphorylation-blocked ERα S216A KI mice, it is now demonstrated that, after being activated by PB, CAR binds and recruits ERα onto the *Sulte1* promoter for subsequent phosphorylation at Ser216. This phosphorylation tightens CAR interacting with ERα and to activates the promoter. Hepatic SULT1E1 mRNA levels are constitutively up-regulated in type 1 diabetic Akita mice; CAR spontaneously accumulates in the nucleus and activates the *Sult1e1* promoter by recruiting phosphorylated ERα in the liver as observed with PB-induced livers. Thus, this CAR-phosphorylated ERα signaling enables these two nuclear receptors to communicate, activating the *Sult1e1* gene in response to either PB or diabetes in mice. ERα phosphorylation may integrate CAR into estrogen actions, providing insights into understanding drug-hormone interactions in clinical therapy.

## Introduction

Estrogens are known to regulate hepatic metabolism and metabolic syndrome besides profound actions in basic biology and disease developments^1–3^. Estrogen is inactivated by sulfation; estrogen sulfotransferase SULT1E1, a member of the cytosolic sulfotransferase superfamily, is the major enzyme that specifically and effectively sulfates estrogen^4–9^. SULT1E1 is expressed low in the liver of normal adult mice but is induced by treatment with therapeutic drugs such as phenobarbital (PB). Nuclear receptor CAR (NR1I3) mediates this induction by activating the *Sult1e1* gene^10^. In addition, SULT1E1 expression is spontaneously up-regulated in the liver of diabetic mice^11^. The molecular mechanism of this spontaneous activation of the *Sult1e1* gene remains unknown. Ablation of the *Sult1* gene affected on diabetic phenotypes in mice, demonstrating that SULT1E1 plays a role in regulating hepatic metabolic syndrome^12^. Here, it was examined whether estrogen receptor α (ERα) regulates the *Sult1e1* gene and whether CAR plays a role in this regulation in diabetic livers. In either induced or spontaneous activation of the *Sult1e1* gene, the molecular mechanism by which CAR and ERα communicate to activate the gene may be conserved.

CAR was found to repress gluconeogenic genes in mouse livers, providing mechanistic insights into understanding the improvement of hepatic insulin sensitivity and the decrease of blood glucose levels in PB-treated epileptic patients^13^. CAR is inactivated by phosphorylation at Thr38 within the DNA binding domain (DBD)^14^. PB and insulin antagonize each other to regulate CAR activation through this phosphorylation^15^. PB stimulates dephosphorylation to activate CAR, whereas insulin represses it to inactivate CAR^16^. Therefore, phosphorylation of CAR is the intersection where drugs and hormones can interfere with each other’s actions. Thr38 of CAR directs its sidechain towards a surface of the ligand-binding domain (LBD), but not towards an interface with DNA^17,18^. This residue regulates both intra- and inter-molecular interactions; phosphorylation of T38 prevents CAR from forming intra-molecular interactions between the DBD and LBD and enables CAR to homodimerize, while dephosphorylation leads to CAR monomers, allowing it to heterodimerize with RXRα (NR2B1). CAR utilizes two different surfaces located on opposite sides of the CAR molecule to form either homodimer or heterodimer. The presence of two dimer interfaces can confer the CAR of the CAR-RXRα heterodimer capability of interacting with an additional nuclear receptor. A recent study shows that such an interaction may occur with RORα (NR1F1) in mouse livers^19^. Two dimer interfaces provide CAR with a structural basis to diversify its interactions with other nuclear receptors which may include ERα, and phosphorylation allows CAR to regulate these interactions^16^.

When ERα, CAR or SULT1E1 was ablated in mice, their livers developed similar metabolic disorders and affected on diabetic phenotypes^11,20,21^, suggesting that the *Sult1e1* gene may be a common target of CAR and ERα, thus these two nuclear receptors may directly interact to regulate the gene. Because the sequence around Thr38 is a conserved phosphorylation motif among most of mouse and human nuclear receptors^16^, regulation by this motif could be extended to many other nuclear receptors. In fact, when CAR interacted with RORα to activate the *Sult1e*1 gene, RORα was phosphorylated at Ser100, the corresponding residue within the DBD^19^. The same motif is conserved in ERα of human (Ser212) and mouse (Ser216)^22,23^. Therefore, it is possible that CAR interacts with ERα through its phosphorylation at Ser216 to activate the *Sult1e1* gene may undergo the same regulation.

Here, we examined CAR-ERα signaling as a mechanism that integrates signals to activate the *Sult1e1* gene in PB-induced as well as diabetic livers. We utilized ERα S216A KI and ex3-ERα KO mice to investigate phosphorylation of Ser216, CAR KO mice to determine the role of CAR in the interaction with phosphorylated ERα and, diabetic Akita and *ob/ob* mice to examine SULT1E1 expression in diabetic livers. A phospho-Ser216 peptide antibody was used to detect phosphorylation of ERα at Ser216. Real-time PCR determined SULT1E1 mRNA levels and chromatin immunoprecipitation assays examined bindings of nuclear receptors to the *Sult1e1* promoter. Interactions and complex formation of nuclear receptors were examined by gel-shift and co-immunoprecipitation assays. First, the mechanism of this CAR-ERα signaling was determined in PB-treated mouse livers and subsequently, examined in diabetic livers. Here, experiments that establish CAR-ERα signaling will be presented and implication of this signal in diabetes will be discussed. Current findings with phosphorylation of the conserved motif should be extended to investigate nuclear receptor communication far beyond CAR.

## Results

### PB induction of SULT1E1 in mouse livers

**Figure 1:**
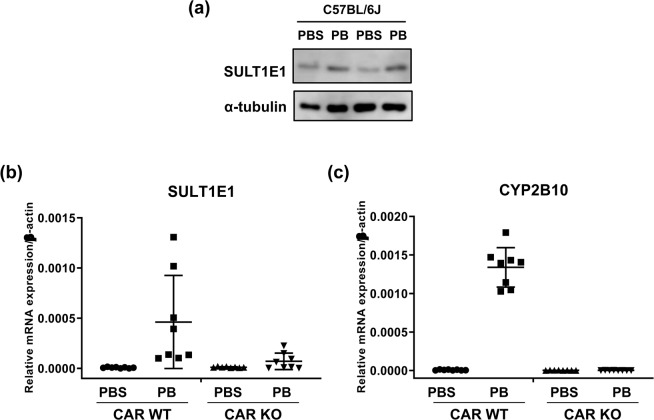
PB-induced expression of SULT1E1 in CAR WT and CAR KO males. (**a**) Cytosolic extracts were prepared from C57BL/6J male livers which were treated with PB or PBS. SULT1E1 was induced by PB-treatment, and a polyclonal antibody for α-tubulin was used as a loading control. (**b**) PB induction of SULT1E1 mRNA in CAR wild-type and KO mouse livers treated with PBS or PB (*N* = 8). *P*-value derived from ANOVA is 0.0013. (**c**) Hepatic RNAs were prepared from CAR WT (*N* = 8) and CAR KO (*N* = 8) males treated with PB or PBS for 24 h. The same RNAs used for SULT1E1 were utilized to measure the levels of CYP2B10 mRNA. All *p*-values for four different groups are lower than 0.0001. All data are presented as means ± S.D. of values of individual mice, and one-way ANOVA was used as a statistical analysis for multiple groups.

Cytosolic fractions were separately prepared from livers of two male mice treated with PBS or PB for 24 h and subjected to western blot analysis. SULT1E1 protein levels were increased after PB treatment in both samples (Fig. 1a and Supplement Fig. 1). Liver RNAs were prepared from CAR WT and CAR KO males treated with PBS or PB for 24 h and subjected to real time PCR analysis. SULT1E1 mRNAs were detected low in PBS-treated livers (Fig. 1b). While PB treatment increased hepatic SULT1E1 mRNA about 58-fold in CAR WT males, this increase was profoundly diminished in CAR KO males (Fig. 1b). Confirming well-known observations^24^, CYP2B10 mRNA was induced in CAR WT over 180-fold but not CAR KO males (Fig. 1c). These observations indicated that CAR activates *the Sult1e1* gene in response to PB. On the other hand, higher residual levels of SULT1E1 mRNA in PB-treated CAR KO compared with that of CYP2B10 mRNA also suggested the presence of an additional factor that regulates the *Sult1e1* gene in the absence of CAR.

### SULT1E1 expressions in ERα KI and KO males

**Figure 2:**
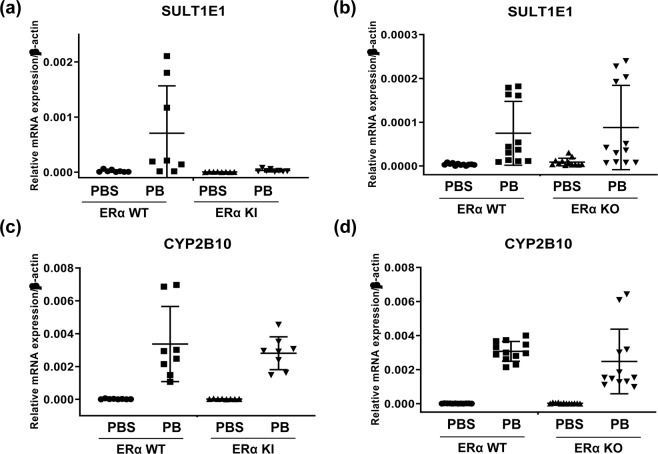
PB-induced expression of SULT1E1 mRNA in ERα S216A KI and ERα KO males. Hepatic RNAs were prepared from (**a**) ERα WT (*N* = 8), and ERα S216A KI (*N* = 8), males or (**b**) ERα WT (*N* = 12) and ERα KO (*N* = 12) males treated with PB or PBS for 24 h, to measure each SULT1E1 mRNA. Each *p*-value of both data set is derived from as 0.0059, and <0.0001, respectively. The same RNA samples from (**c**) ERα WT (*N* = 8), and ERα S216A KI (*N* = 8), males or (**d**) ERα WT (*N* = 12) and ERα KO (*N* = 12) males were used to measure CYP2B10 mRNA. The both *p*-values of data are estimated as lower than 0.0001. All data are presented as means ± S.D. of values of individual mice. One-way ANOVA was used as a statistical analysis for data set of ERα S216A KI, and Kruskal-Wallis test was used to analyze the data set of ERα KO.

ERα WT and ERα S216A KI male mice were treated with PBS or PB, and RNAs were prepared for real time PCR analysis (Fig. 2a). SULT1E1 mRNA levels were increased about 33-fold by PB treatment in ERα WT males, whereas this increase was severely repressed in the ERα S216A KI males. These observations suggested that ERα might be phosphorylated at Ser216 when ERα is present to activate the *Sult1e1* gene. Unexpectedly, SULT1E1 mRNA was equally induced in ERα WT and ERα KO males (Fig. 2b), suggesting that PB-activated CAR can initiate SULT1E1 transcription in the absence of ERα. In contrast to SULT1E1, CYP2B10 mRNA was induced over 155-fold and 125-fold by PB in ERα S216A KI and KO males, respectively (Fig. 2c,d), demonstrating that ERα was not involved in PB induction of the *Cyp2b10* gene.

### Phosphorylated ERα binding the *Sult1e1* promoter

**Figure 3:**
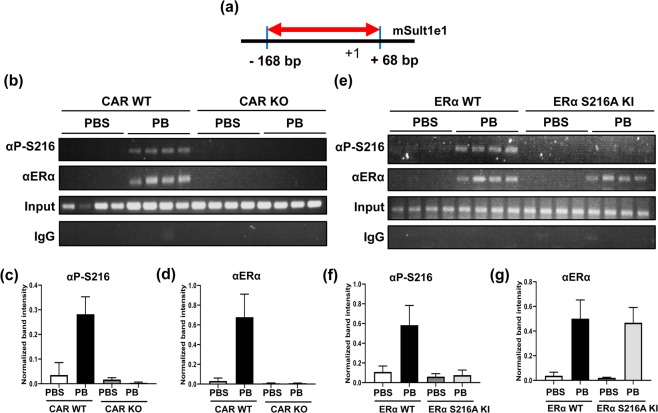
PB-induced binding of ERα phosphorylated at Ser216 to the *Sult1e1* promoter in male mice. ChIP assays were employed to show ERα bindings to a proximal *Sult1e1* promoter. (**a**) Schematic representation of a region that was amplified and primers that were used for PCR amplification. (**b**) Chromatins were prepared from livers of CAR WT and KO males treated with PB or PBS for 6 h. And those were subjected to ChIP assays with either an ERα antibody or a P-S216 peptide antibody. Both of (**c**,**d**) show the quantification, which was normalized by input intensity, for P-ERα and ERα respectively. The densitometry was performed by ImageJ. Kruskal-Wallis test and one-way ANOVA were used as statistical analyses, and *p*-values are 0.0004 and <0.0001 respectively. (**e**) Chromatins were prepared from livers of ERα and KI males treated with PB or PBS for 6 h. And those chromatins were subjected to ChIP assays with either an ERα antibody or a P-S216 peptide antibody. Rabbit IgG was used as a negative control. Both of (**f**,**g**) present the quantifying data from ERα and KI males, which was normalized by input intensity, for P-ERα and ERα respectively. The densitometry was performed by ImageJ. Kruskal-Wallis test and one-way ANOVA were used as statistical analyses, and *p*-values are 0.0064 and <0.0001 respectively. All data are presented as means ± S.D. of values of individual mice.

Chromatins were prepared from the liver of male mice treated with PBS or PB for subsequent chromatin immunoprecipitation (ChIP) assays. For this assay, a 236 bp (−168/+68) sequence within the proximal promoter was amplified (Fig. 3a). We found increased ERα binding to the *Sult1e1* promoter, and ERα appeared to be bound to the promoter following PB treatment. It was phosphorylated at Ser216 in CAR WT males; this binding and phosphorylation was not observed in CAR KO males (Fig. 3b–d, and Supplement Fig. 2a). On the other hand, ERα bound the promoter but was not phosphorylated at Ser216 in ERα S216A KI males (Fig. 3e–g, and Supplement Fig. 2b). These observations first demonstrated the high specificity of this αP-S216 antibody to phosphorylated ERα at Ser216 in ChIP assays. Moreover, the finding with CAR KO males indicated that CAR is required for ERα to bind the promoter and for subsequent phosphorylation; ERα binds the promoter first and then, subsequently, is phosphorylated at Ser216.

### A CAR-phosphorylated ERα complex on the promoter

**Figure 4:**
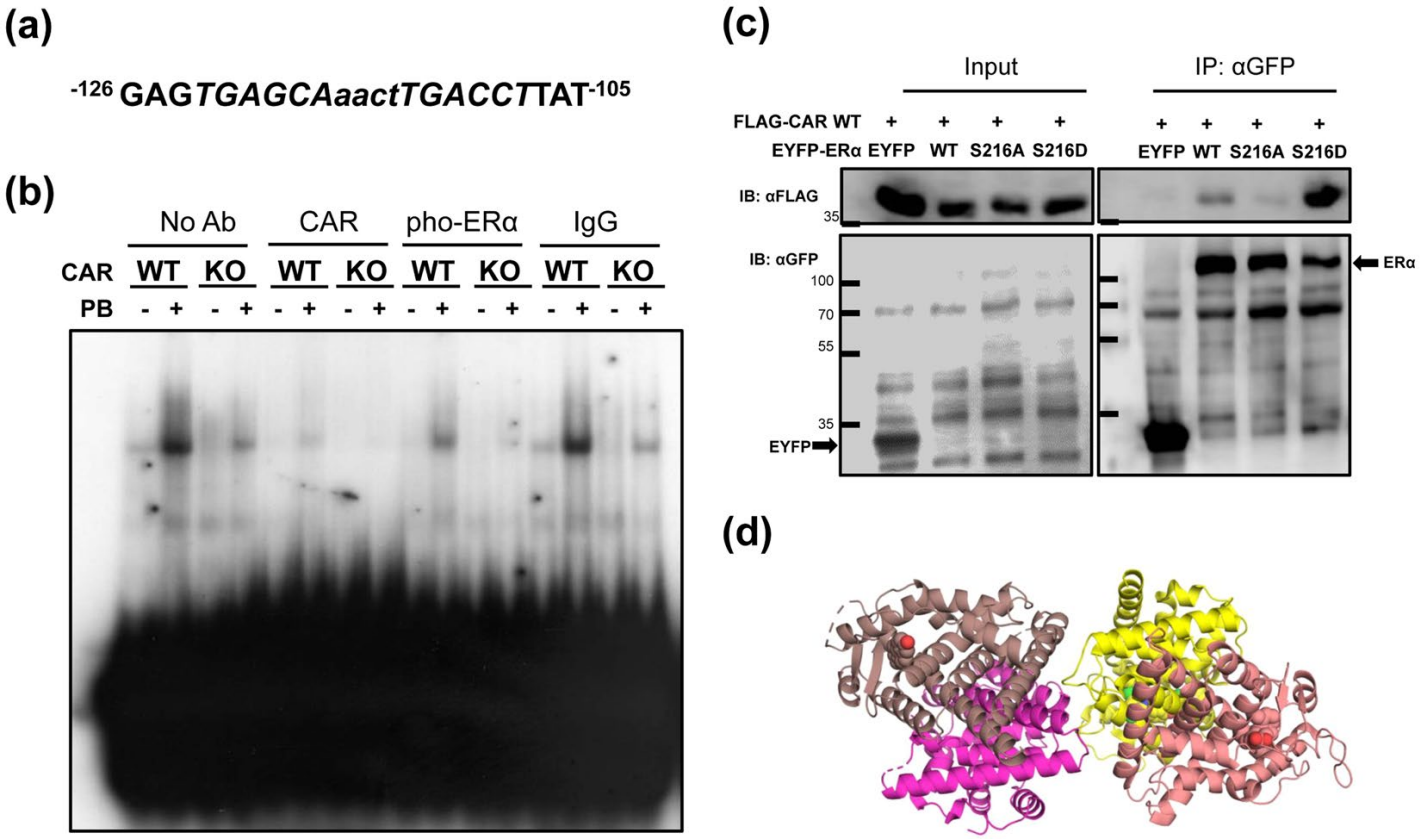
Interactions between CAR and phosphorylated ERα. (**a**) The DR4 sequence was used as a probe for gel shift assays. (**b**) ^32^P-labeled double stranded probe was mixed with nuclear extracts prepared from mouse livers as indicated in the figure. For super shifts, CAR or phosphorylated ERα antibody was added to a mixture of probe and nuclear extract as indicated in the figure. (**c**) FLAG-tagged CAR was co-expressed with either EYFP-tagged ERα WT, ERα S216A, and ERα S216D in Huh7 cells in the presence of 10 nM 17β-estradiol for 24 h. Whole cell lysates were isolated and precipitated with αGFP-resin, eluted proteins from which were loaded on a SDS-PAGE gel and subjected to staining with an αFLAG antibody. Full-length gel images are described as Supplement Fig. 3. (**d**) 3D model of the hypothetical heterotetramer between an ERα homodimer (monomers in brown and magenta) and the CAR/RXRα heterodimer (CAR in yellow and RXRα in ash-rose). In this model one surface of CAR interacts with RXRα, while another surface interacts with an ERα subunit. Ligands (17β-estradiol (ERα), 9-*cis*-retinoic acid (RXRα), 3,5-dichloro-2-(4-[(3,5-dichloropyridin-2-yl)oxy]phenoxy)pyridine (CAR)) are shown with atoms in spheres (oxygen in red, nitrogen in blue and chlorine in green, carbon atoms are colored as protein the ligands are bound to). This nuclear receptor tetramer may comprise a complex which regulates the expression of *Sult1e1* gene. A description of how the model tetramer was created can be found in the Methods.

The DR4 within the 263 bp sequence of the promoter (Fig. 4a) was used for gel shift assays with nuclear extracts prepared from the liver of CAR WT and CAR KO males treated with PBS or PB (Fig. 4b). PB treatment profoundly increased a disappeared band in the extract from CAR WT males. A corresponding band was neither detected nor increased in that from CAR KO males. This band formation was inhibited by adding an anti-CAR or an αP-S216 antibody to reaction mixtures. These observations suggested that a complex containing both CAR and phosphorylated ERα binds the DR4 probe. Subsequently, coimmunoprecipitation assays were employed to examine interactions between CAR and ERα. Flag-tagged CAR was co-expressed with EYFP-tagged ERα WT, ERα S216A or ERα S216D in Huh7 cells in the presence of 10 nM 17β-estradiol. Precipitates formed by an anti-GFP antibody were analyzed by western blot using an anti-Flag antibody (Fig. 4c). CAR was effectively co-precipitated with ERα S216D, indicating that CAR forms a strong complex with phosphorylated ERα (Fig. 4c). In the absence of estrogen, this coprecipitation was not observed (data not shown). A 3D model of ERα homodimer interacting with CAR-RXRα heterodimer was created based on the homodimer of CAR. (Fig. 4d). This nuclear receptor heterotetramer may comprise a complex which regulates the expression of *Sult1e1* gene.

### SULT1E1 expression in diabetic livers

**Figure 5:**
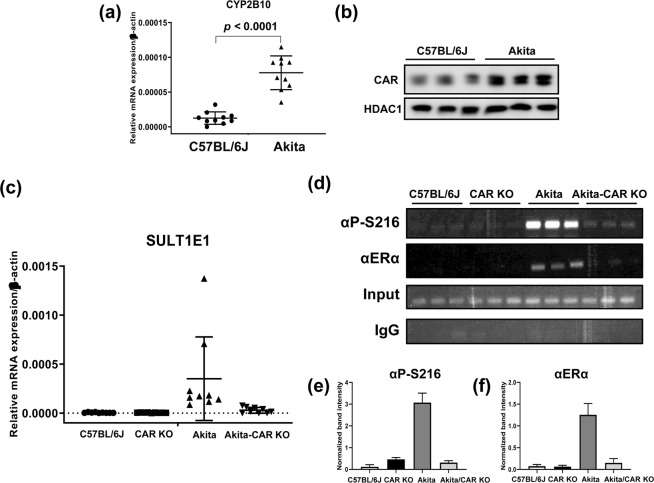
Hepatic SULT1E1 expression in Akita-CAR KO males. (**a**) Hepatic RNAs were prepared from C57BL/6J (*N* = 10) and Akita (*N* = 10) males. CYP2B10 mRNA, the classic CAR target showed the higher expression in Akita compared to C57BL/6J. Data set was analyzed by a Student’s *t*-test, and *p*-value is lower than 0.0001. (**b**) Nuclear extracts were separately prepared form livers of C57BL/6J (*N* = 3) and Akita (*N* = 3). The expression of CAR in nucleus was higher than that of C57BL/6J. (**c**) Hepatic RNAs were prepared from CAR WT (*N* = 9), CAR KO (*N* = 8), Akita (*N* = 9) and Akita-CAR KO (*N* = 6). Data set was analyzed by Kruskal-Wallis test, and *p*-value is 0.0001. (**d**) Chromatins were separately prepared from three livers of each of the above-mentioned groups for subsequent ChIP assays. Both of (**e,f**) presented the quantifying data, which were normalized by input intensity, for P-ERα and ERα respectively. The densitometry was performed by ImageJ. All data are presented as means ± S.D. of values of individual mice and one-way ANOVA was used as a statistical analysis. Each *p*-value of both groups is lower than 0.0001.

The C57BL/6J-derived Akita mice inherit the mutated insulin 1 gene and are used as an animal model for type 1 diabetes. CYP2B10 mRNA levels were up-regulated and CAR spontaneously accumulated in the nucleus in Akita males compared with those in C57BL/6J mice (Fig. 5a,b and Supplement Fig. 4). Levels of SULT1E1 mRNA were also up-regulated in the liver of Akita relative to those in C57BL/6J and CAR KO mice (Fig. 5c). To examine whether CAR regulated the expression of SULT1E1 in Akita, the *Car* gene was deleted from Akita by crossing Akita with CAR KO mice. The up-regulation of the basal of SULT1E1 mRNA expressions was no longer detected in Akita/CAR KO mice (Fig. 5c). ChIP assays were performed to examine ERα binding to the *Sult1e1* promoter (Fig. 5d–f, and Supplement Fig. 5). ERα was only found with Akita, but with neither C57BL/6J, CAR KO nor Akita/CAR KO mice. Moreover, this ERα was phosphorylated at Ser216. Thus, the CAR-phosphorylated ERα signal regulated the promoter activation in Akita livers. Similar, the up-regulation of CYP2B10 mRNA and promoter binding of phosphorylated ERα were also observed in the livers of *ob/ob* mice, an animal model of type 2 diabetes (Supplement Fig. 6).

### ERα interactions with RORα

**Figure 6:**
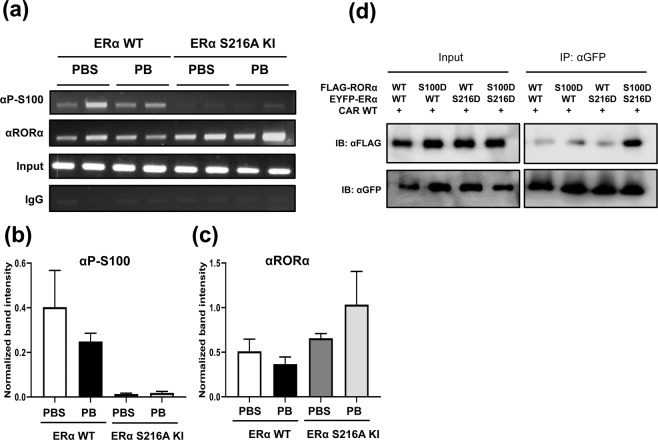
Non-phosphorylated ERα is not bound to the *Sult1e1* promoter in male mice with P-RORα. ChIP assays were applied to show RORα bindings to a proximal *Sult1e1* promoter. (**a**) Chromatins were prepared from livers of ERα WT and KI males which were subjected to ChIP assays with either an RORα antibody or a P-S100 peptide antibody. Both of (**b**,**c**) indicate the data quantification, which was normalized by input intensity, for P-RORα and RORα respectively. The assay was examined by ImageJ, and one-way ANOVA were used as statistical analyses, and *p*-values are 0.0247 and 0.1080 respectively. (**d**) V5-CAR WT (no tag) was co-expressed with FLAG-tagged RORα WT, RORα S100D, EYFP-tagged ERα WT, and ERα S216D in Huh7 cells. Whole cell lysates were isolated and precipitated with αGFP-resin, and eluted proteins were loaded on a SDS-PAGE gel and subjected to staining with an αGFP- or αFLAG antibody.

RORα repressed the *Sult1e1* gene by binding its proximal promoter in mouse livers. In response to PB, RORα was phosphorylated at Ser100, remaining on the promoter and co-activating it in CAR WT but not CAR KO mice^19^. In order to examine as to how ERα played a role in this regulation of RORα ChIP assays were employed using a phospho-Ser100 peptide antibody and ERα KI mice. As observed in our previous work^19^, RORα was found to bind the promoter before PB treatment in the liver of ERα KI mice (Figs. 6a,c, and Supplement Fig. 7). While RORα became phosphorylated at Ser100 in ERα KI WT mice, this phosphorylation was not observed in ERα KI mice (Fig. 6a,b, and Supplement Fig. 7). It was appeared that RORα phosphorylation required ERα to be phosphorylated on the promoter. Moreover, ERα and RORα may have interacted when both were phosphorylated. Consistent with notion, Co-IP assays showed that ERα S216D and RORα S100D were most effectively co-precipitated (Fig. 6d and Supplement Fig. 8).

## Discussion

Nuclear receptors have the conserved phosphorylation motif within their DNA binding domains^16^. Phosphorylation of this motif enables nuclear receptors to interact among each other, thereby integrating their functions. Mouse ERα has this motif at Ser216^23^, which becomes phosphorylated on the *Sult1e1* promoter in PB-treated and diabetic mouse livers. CAR recruits ERα onto the *Sult1e1* promoter for subsequent phosphorylation of Ser216, thereby strengthening its interaction with ERα and activating the promoter. Thus, phosphorylation enables CAR to communicate with ERα and integrates it into CAR-mediated activation of the gene.

**Figure 7:**
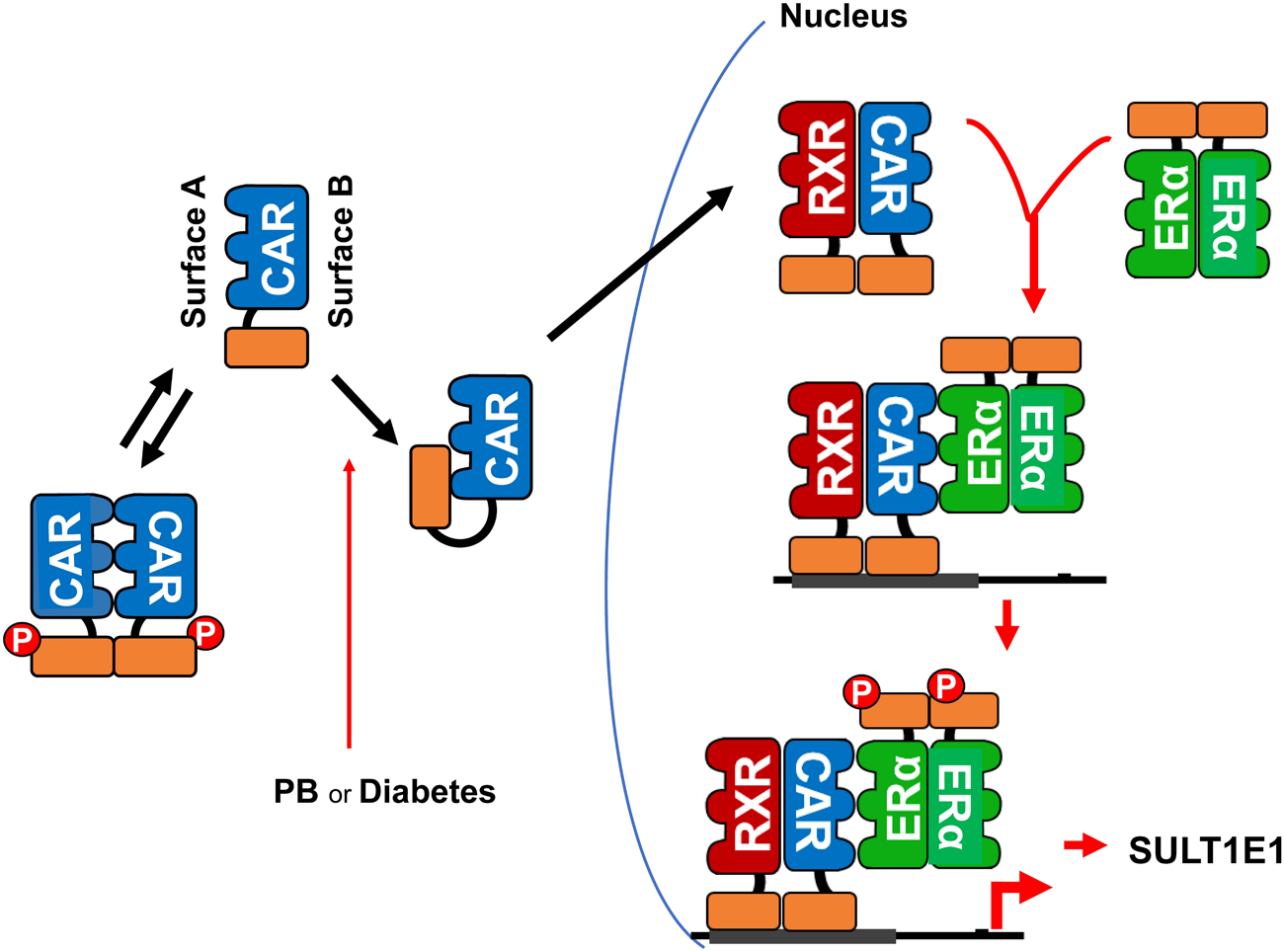
Schematic depiction of the CAR-ERα communication mechanism. CAR phosphorylated at Thr38 forms a homodimer through surface A and remains inactive in the cytoplasm^14,17^. In response to PB, Thr38 is dephosphorylated and non-phosphorylated CAR homodimer dissociates, allowing the interaction between the DBD and LBD to translocate into the nucleus^17,18^. In the nucleus, CAR utilizes its surface B to form an RXRα-CAR heterodimer. ERα dimerizes through B surfaces upon estrogen binding. Subsequently, RXRα-CAR heterodimer recruits ERα homodimer to the *Sulte1* promoter by between the heterodimer-bound CAR and homodimer-bound ERα *via* their A surfaces. While phosphorylated ERα is unable to directly bind DNA, its homodimer sandwiches RXRα-CAR heterodimer and phosphorylated RORα, resulting in an activation of the promoter.

CAR forms a heterodimer with RXRα through a well-known B dimer interface and is expected to utilize the A dimer interface to interact with ERα (Fig. 7). ERα forms a homodimer through its B surfaces in the X-ray crystal structure^25^, leaving A surfaces of both subunits to interact with another nuclear receptor such as CAR. In this model of interactions, CAR from the RXRα-CAR heterodimer and one monomer of the ERα homodimer interact through their A surfaces (Fig. 4d). CAR initiates formation of this complex on the *Sult1e1* promoter before ERα is phosphorylated. Whereas this complex with non-phosphorylated ERα formed in ERα S216A KI mice, the *Sult1e1* gene was not activated, indicating that it is an inactive transcription complex. Phosphorylation of Ser216 occurs subsequent to formation of this inactive complex on the promoter, converting this inactive form to an active transcription complex. In fact, our Co-IP experiment showed that that the phosphorylation greatly enhances interactions between CAR and ERα, supporting this notion. The uniqueness of this mechanism is that whole reaction processes from complex formation to phosphorylation and to activation undergo sequentially on the promoter, which enables CAR and ERα to target the *Sult1e1* promoter and regulate it specifically and effectively.

CAR was previously shown to form a complex with RORα on the *Sult1e1* promoter in mouse livers in response to PB treatment^19^. Upon treatment, RORα, which repressed the promoter, became phosphorylated at Ser100, the corresponding conserved phosphorylation motif within the DBD. This phosphorylation converted RORα from a corepressor to co-activator for CAR to activate the promoter. Therefore, CAR appears to integrate RORα in addition to ERα through phosphorylation of their conserved motifs. Our present study found that this conversion did not occur in ERα KI mice and, that ERα and RORα strongly interacted when both were phosphorylated. These observations are consistent with the hypothesis that phosphorylated ERα interacts with RORα for subsequent phosphorylation, thereby converting RORα to an activator. For this end, since phosphorylated ERα interacted with CAR and phosphorylated RORα, a phosphorylated ERα homodimer may sandwich CAR and phosphorylated RORα. The molecular mechanism of how these three nuclear receptors coordinate to regulate the *Sult1e1* gene remains to be investigated in the future.

PB treatment is known to decrease blood glucose levels and improve insulin sensitivity in epileptic patients^26^. Similar to insulin but *via* different mechanism, PB represses hepatic gluconeogenesis, causing beneficial effects against the development and progression of type 2 diabetes^27^. Insulin excludes FOXO1 from the nucleus while PB enables CAR to directly interact with and inactivate FOXO1, both resulting in the repression of FOXO1-mediated activation of gluconeogenic genes. On the other hand, insulin antagonizes PB to stimulate ERK1/2 signal, which retains inactive CAR in the cytoplasm. When insulin actions are deteriorated in diabetic livers, CAR spontaneously accumulates in the nucleus. Either by PB or spontaneous activation, CAR recruits phosphorylated ERα to activate the *Sult1e1* gene. Therefore, these observations a consistent with the notion that an increase of SULT1E1 enzyme produces beneficial responses against diabetes. Estrogen is an anti-inflammatory hormone^28,29^ which causes both beneficial and toxic effects^30^, SULT1E1 may balance estrogen effects by metabolizing estrogen. Ablation of the *Sult1e1* gene increased susceptibility to LPS-induced sepsis in mice^11^. Moreover, hepatic diabetic phenotype was worsened by ablation of *the Sult1e1* gene in male *ob/ob* mice^12^. Future studies may establish the CAR-phosphorylated ERα pathway as the signal pathway that regulates active estrogen levels to counter pathophysiological developments such as diabetes.

In conclusion, CAR is the principle transcription factor that activates the *Sult1e1* gene by recruiting ERα phosphorylated at Ser216 to activate the *Sult1e1* gene. Ser216, the conserved phosphorylation motif within the DBD, is utilized by CAR to communicate with ERα, integrating it into CAR-mediated gene’s activation. Ser216 is conserved in the majority of nuclear receptors such as Ser100 of RORα; as CAR does with ERα and also RORα, nuclear receptors may communicate among themselves through the conserved motif to increase and diverge their actions.

This phosphorylation-medicated communication of CAR with ERα is also utilized to activate the *Sult1e1* gene in diabetic livers, possibly allowing us to develop drugs that target phosphorylated ERα for clinical therapy.

## Methods

### Reagents and plasmids

Polyclonal antibodies for SULT1E1 (12522-1-AP) and α-tubulin (#2144S) were manufactured by ProteinTech (Rosemont, IL, USA) and Cell Signaling Technology (Beverly, MA, USA), respectively. Monoclonal antibodies against ERα, ab32063 and sc-543 were obtained from Abcam Plc. (Cambridge, UK) and Santa Cruz Biotech, Inc. (Dallas, TX, USA), respectively. Each monoclonal antibody against CAR (PP-N4111-00) and RORα (PP-H3910-00) was produced by Perseus Proteomics (Tokyo, Japan). Polyclonal antibodies against RXR (sc-774), HDAC1 (sc-6298), normal rabbit IgG (sc-2027), each secondary antibody of mouse (sc-2314) and goat (sc-2020) were purchased from Santa Cruz Biotech, Inc. (Dallas, TX, USA). An antibody for GFP-HRP (ab6663) was obtained from Abcam (Cambridge, UK). A HRP-conjugated anti-FLAG M2 (A8592) was from Sigma-Aldrich (St. Louis, MO, USA). Anti-phospho-Ser216 peptide antibodies (αP-Ser216) for mERα and hRORα (αP-Ser100) were generated by GenScript (Piscataway, NJ, USA). An anti-phospho-Ser212 peptide antibody for hERα was produced by Anaspec (Fremont, CA, USA). A HRP-conjugated secondary antibody of rabbit (65-6120) was purchased from Thermo Fisher Scientific (Waltham, MA, USA). A FuGENE 6 transfection reagent (E2691) was from Promega Corporation (Madison, WI, USA). Bio-Rad Protein Assay reagent was from Bio-Rad (Hercules, CA, USA). WesternBright ECL and Sirius HRP substrate were from Advansta (San Jose, CA, USA). Restriction enzymes were from New England Biolabs (Ipswich, MA, USA), and the ChIP IT Express kit was manufactured from Active Motif (Carlsbad, CA, USA). pGL3 vectors harboring mouse *Sult1e1* promoter were constructed as described in our previous study^31^. All constructs were verified by nucleotide sequencing.

### Animals

ERα WT and Ex3-ERα KO mice were kindly provided by Drs. Sylvia Hewitt and Kenneth Korach at NIEHS^32^. ERα S216A KI mice were generated by the core at NIEHS, and they are fertile. More details for characterization of this mouse strain will be published elsewhere. Briefly, DNA fragments of the *Esr1* gene of C57BL/6J genes were mutated and injected ES cells from 129 mice. Brothers and sisters generated heterozygous were crossed, form which ERα WT and ERα KI lines were established. Both males and males are fertile. CAR KO mice in C57BL/6J background were produced in house. Akita and *ob/ob* mice were purchased from Jackson Laboratory. Akita heterozygotes were crossed with CAR KO mice in house and genotyped using proper probes produced by Transnetyx (Cordova, TN, USA), to ablate CAR (Akita-CAR KO). PB (100 mg/kg body weight) in PBS or PBS was intraperitoneally injected into 8- to 15-week-old male mice for 6 or 24 h. Each mouse was maintained under the standard condition at the NIEHS. Animal experiments were conducted per protocols approved by the Animal Care and Use Committee (ACUC) at NIEHS/NIH. All methods and procedures were performed in accordance with the Public Health Service Policy with humane examinations.

### Extraction of RNA and quantitative real-time PCR (RT-PCR)

Total RNAs from mouse livers were isolated using TRIzol reagent (Invitrogen, Carlsbad, CA, USA) according to the manufacturer’s instructions. All of isolated RNAs were quantified and certified using NanoPhotometer (Implen GmbH, München, Germany). Each cDNA was synthesized with High Capacity cDNA Reverse Transcription Kit (Thermo Fisher Scientific, Waltham, MA, USA) adding 2 µg of total RNA to the reaction mixture, and the reaction mixtures were incubated at 37 °C for 2 h. Each synthesized cDNA was quantified and certified using Beckman DU 640 spectrophotometer (Beckman Coulter Inc., Brea, CA, USA) by measuring the absorbance at 260 nm and 280 nm. Taqman probes were used as Mm00499178_m1, Mm00456591_m1 and REF4352663 for SULT1E1, CYP2B10 and β-actin, respectively. qPCR was performed with the CFX96 Touch Real-Time PCR Detection System (Bio-Rad Laboratories Inc., Hercules, CA, USA). Each value was derived from the comparative CT method which compared the *C*_t_ value of one target gene to reference gene using the 2^−ΔΔ*C*t^ formula according to the manufacturer’s guideline. Δ*C*_t_ indicates the differences in threshold cycles for target and reference (*C*_t,target_ − *C*_t,reference_), and ΔΔ*C*_t_ represents the relative change in these differences between the target and reference (Δ*C*_t,target_ − Δ*C*_t,reference_). Therefore, the expression of target, normalized to a house keeping gene, was given by 2^−ΔΔ*C*t^.

### Extraction of protein and western blot

The cytosolic and nuclear proteins from the mouse livers were prepared by NE-PER nuclear and cytoplasmic extraction reagents (78835, ThermoFisher Scientific) based on the manufacturer’s instruction. About 20–50 mg liver was cut into small pieces and washed with PBS. The tissue pellet was homogenized by a Dounce homogenizer in the 500 µL volume of CER I reagent. Each tube was vortexed vigorously for 15 sec to fully suspend the pellet, and incubated on ice for 10 min. Then, the 27.5 µL of pre-chilled CER II reagent was added into a tube, mixed thoroughly, incubated on ice for 1 min and centrifuged for 5 min at 12,000 × *g*. The supernatant containing cytoplasmic extract was transferred to a pre-chilled tube, and the remain insoluble fraction was suspended into the 250 µL of NER reagent. After 15 sec of thorough mixing, a tube was placed on ice and continued vortexing for 15 sec every 10 min. After a total of 40 min, all of samples were centrifuged at 12,000 × *g* for 10 min, and the supernatant fraction which contained nuclear extract was transferred to a clean pre-chilled tube. All prepared proteins were stored at −80 °C and thawed in ice before using. Huh7 cells lysates were extracted using IP buffer containing 20 mM Tris-HCl (pH 7.5), 250 mM NaCl, 1% (v/v) Triton-X 100, 10% (v/v) glycerol, protease inhibitors and phosphatase inhibitors. Each protein concentration was estimated using Bio-Rad protein assay dye reagent. Each sample was separated into a 10% SDS-PAGE gel, and separated proteins were transferred onto a PVDF membrane (GE Healthcare, Little Chalfont, UK). The nonspecific proteins were blocked using 5% (w/v) skim milk or 5% (w/v) BSA in tris-buffered saline containing 0.1% (v/v) Tween 20 (T-TBS). A WesternBright ECL or Sirius kit (Advansta Inc., San Jose, CA, USA) detected HRP substrates, and results were visualized by a C-DiGit Chemiluminescent Western Blot Scanner (LI-COR, Inc., Lincoln, NE, USA). Quantitative densitometry was determined using ImageJ 1.52a, Java 1.8.0_112 version (NIH, Bethesda, MD, USA). Values are means ± S.D relative to each described loading control.

### Chromatin immunoprecipitation assay (ChIP)

About 200 mg liver was minced and incubated in a 1% (v/v) formaldehyde for 10 min followed by a glycine incubation for 5 min at room temperature before homogenization^19^. Each suspension which contained nuclear protein was obtained by filtering homogenates through a 100 μm of Falcon cell strainer (BD Biosciences, Bedford, MA, USA)^19^, incubated in the lysis buffer, and sonicated in the shearing buffer including 500 μM of PMSF and PIC as manufacturer’s guide line (Active motif). Immunoprecipitations were performed overnight with an ERα antibody or a phospho-ERα antibody respectively using protein G conjugated magnetic beads. The beads were washed with ChIP buffers, chromatins were eluted, and the cross-linking was reversed prior to PCR (38 cycles) amplification using primers targeting the proximal region (−168/+68) of mouse *Sult1e1* promoter. The sequence of primer pairs is: forward primer 5′ACCCAAAGGGGAGAAACAGC-3′, and reverse primer 5′-TCGAATGGCAGCACGATTCT-3′.

### Electrophoretic mobility gel shit assay (EMSA)

Gel mobility shift assays were performed as described in previous studies^19,33^. Briefly, annealed double-strand oligonucleotides were labelled with [α-^32^P]dATP by fill-in reaction using T4 polynucleotide kinase (PNK) to generate radioactive probes. For observing the supershifts, mixtures were incubated with 1 µg of nuclear protein extracts and anti-phospho ERα or -CAR antibodies at room temperature for 10 min. Nucleotide sequences used as probes are described in Fig. 4a.

### Cell culture and transfection

Huh7 (hepatocyte-derived carcinoma cell line) was purchased from the American Type Culture Collection (ATCC; Manassas, VA, USA). Dulbecco’s modified eagle medium (DMEM; 11965-092) was manufactured by Gibco (Waltham, MA, USA). Fetal bovine serum (FBS; S11150) was produced by Atlanta Biologicals (Flowery Branch, GA, USA). Penicillin-streptomycin (P0781) and sodium pyruvate (S8636) were purchased from Sigma-Aldrich (St. Louis, MO, USA). Huh7 cells were cultured in DMEM which supplemented with 10% FBS (v/v), 1 mM sodium pyruvate, 100 units/mL penicillin, and 100 µg/mL streptomycin at 37 °C containing 5% CO_2_ in the humidified atmosphere. Transfections with each plasmid were performed using FuGENE 6 per the manufacturer’s protocols. For each well, 1 × 10^5^ Huh7 cells were distributed into a 100 mm culture dish and incubated at 37 °C for 24 h. The transfection mixture was prepared with containing 5 µg of each cDNA, 25 µL of FuGENE 6 and 10 mL Opti-MEM reduced serum medium (31985-070, Gibco) at room temperature for 15 min. When the cells turned into adhesive, the growth medium was replaced with the transfection mixture and cells were incubated for additional 24 h at 37 °C.

### Co-immunoprecipitation (Co-IP)

Huh7 cells transfected with indicated plasmids for 24 h were lysed in immunoprecipitation (IP) buffer containing 20 mM Tris-HCl (pH 7.5), 250 mM NaCl, 1% (v/v) Triton-X 100, 10% (v/v) glycerol, protease and phosphatase inhibitor cocktails (78442, ThermoFisher Scientific). Each lysate was collected by centrifugation and sonicated. The collected supernatants were incubated with anti-GFP antibody-conjugated agarose at 4 °C for overnight. Resins were washed three times with the IP buffer, subjected to SDS-PAGE for western blot analysis.

### Model displays a hypothetical heterotetramer of an ERα homodimer with a CAR/RXRα heterodimer

The relative orientation of the ERα homodimer to the CAR/RXRα heterodimer was determined by superimposing the CAR from the CAR/RXRα heterodimer (PDBid:1XLS; CAR in yellow/RXRα in ash-rose Fig. 4d) onto molecule A of the CAR homodimer (PDBid:1XNX; not shown) while a monomer from the ERα homodimer (PDBid:1ERE; magenta/brown) was superimposed onto molecule B of the CAR homodimer (PDBid:1XNX; not shown). Ligands from the respective structures are shown in spheres. Superpositions and graphics were performed using the PyMOL Molecular Graphics System, version 2.0 Schrödinger, LLC. (New York, NY, USA).

### Data analysis

Data were analyzed by D’Agostino-Pearson test for evaluating normality. Data of two groups which meet the assumption of normality were analyzed by Student’s *t*-test. Data of multiple groups which meet the assumption of normality were analyzed by one-way ANOVA test, and which do not meet the assumption were analyzed by Kruskal-Wallis test. All statistical analyses and graphical visualizations were conducted by Prism 8.2.1 (GraphPad Software, San Diego, CA, USA). All data are presented as means ± S.D. of each independent value. *P*-values < 0.05 were regarded as statistically significant.

## Supplementary information

Supplementary information.

## Data Availability

The data sets generated during and/or analyzed in the current study will be made available to the research community upon requests.
