# Supplementary material for: Nuclear receptor CAR-ERα signaling regulates the estrogen sulfotransferase gene in the liver
Source: Sci Rep. 2020 Mar 19;10:5001. doi: 10.1038/s41598-020-61767-9 (PMC7081254; doi:10.1038/s41598-020-61767-9)

Supplementary information

Nuclear receptor CAR-ERα signaling regulates the estrogen sulfotransferase gene in the liver

MyeongJin Yi, Muluneh Fashe, Shingo Arakawa, Rick Moore, Tatsuya Sueyoshi and Masahiko Negishi*

Pharmacogenetics Section, Reproductive and Developmental Biology Laboratory, National Institute of Environmental Health Sciences, National Institutes of Health, Research Triangle Park, North Carolina 27709, USA

Supplement figure 1. Band intensities and full-length gel image of Fig. 1a. (a) The densitometry was quantified using ImageJ, and student *t*-test was used as a statistical analysis. (b) Extracted liver cytosol proteins were immunoblotted with SULT1E1 or (c) α-tubulin antibodies, respectively.

Supplement figure 2. Full-length gel images of Fig. 3b and 3e. The size of amplified PCR product was ~236 bp which targets the proximal region (-168/+68) of mouse *Sult1e1* promoter. (a) Chromatins were prepared from livers of CAR WT and KO males treated with PB or PBS for 6 h, and (b) chromatins were prepared from livers of ERα and KI males treated with PB or PBS for 6 h. IgG indicates a rabbit IgG which was used as a negative control.

Supplement figure 3. Full-length gel images of Fig. 4c. FLAG-tagged CAR was co-expressed with either EYFP-tagged ERα WT, ERα S216A, and ERα S216D in Huh7 cells. (a) Whole cell

lysates were isolated and precipitated with αGFP-resin. Eluted proteins were loaded on a SDS-PAGE gel and subjected to immunoblot with an αFLAG antibody. Left four lanes indicate the input samples which were not precipitated with αGFP. (b) Input samples were subjected to immunoblot with an αGFP antibody. (c) Eluted proteins were immunoblotted with an αGFP antibody. A size marker is included in the first gel of each image.

Supplement figure 4. Band intensities and full-length gel image of Fig. 5b. (a) The densitometry was quantified using ImageJ, and student *t*-test was used as a statistical analysis. (b) Extracted proteins were immunoblotted with CAR or (c) HDAC antibodies, respectively. PBS; saline-treated nuclear fraction prepared from C57BL/6J mouse after 6 hours treatment, PB; PB-treated nuclear fraction prepared from C57BL/6J mouse after 6 hours treatment.

Supplement figure 5. Full-length gel image of Fig. 5d. The size of amplified PCR product was ~236 bp which targets the proximal region (-168/+68) of mouse *Sult1e1* promoter. Chromatins were separately prepared from three livers of each of C57BL/6J, CAR KO, Akita, and Akita-CAR KO for subsequent ChIP assays.

Supplement figure 6. Hepatic SULT1E1 expression and promoter binding of phosphorylated ERα at Ser216 in diabetic type 2 mice. (a) Each hepatic RNAs were extracted from heterozygous *ob/+* (*N* = 20) and homozygous *ob/ob* (*N* = 20) males which were treated with PB or PBS for 24 h. The increase of SULT1E1 mRNA was significant in *ob/ob* mice, and *p*-value from Kruskal-Wallis test is derived as <0.0001. (b) Chromatins were prepared form the same livers and subjected to ChIP assays. Both of (c) and (d) showed the quantification for each amplified band, which was

normalized by input intensity, for P-ERα and ERα respectively. The densitometry was performed by ImageJ and one-way ANOVA was used as a statistical analysis for multiple groups. The significant binding difference was observed in P-ERα (*p*-value 0.0053), but not in ERα (*p*-value 0.7459). All data are presented as means ± S.D. of values of individual mice. (e) Full-length images of PCR data from ChIP assay.

Supplement figure 7. Full-length gel images of Fig. 6a. The size of amplified PCR product was ~236 bp which targets the proximal region (-168/+68) of mouse *Sult1e1* promoter. Chromatins were prepared from livers of ERα WT and KI males which were subjected to ChIP assays with either a P-S100 peptide antibody or an RORα antibody.

Supplement figure 8. Full-length gel images of Fig. 6d. V5-CAR WT (no tag) was co-expressed with FLAG-tagged RORα WT, RORα S100D, EYFP-tagged ERα WT, and ERα S216D in Huh7 cells. Whole cell lysates were isolated and precipitated with αGFP-resin. The input samples were not precipitated with αGFP, and IP samples were precipitated with αGFP. (a) Each protein was loaded on a SDS-PAGE gel and subjected to immunoblot with an αFLAG antibody. (b) Eluted proteins were immunoblotted with an αGFP antibody. A size marker is included in each image.

Supplement figure 1.

(a)

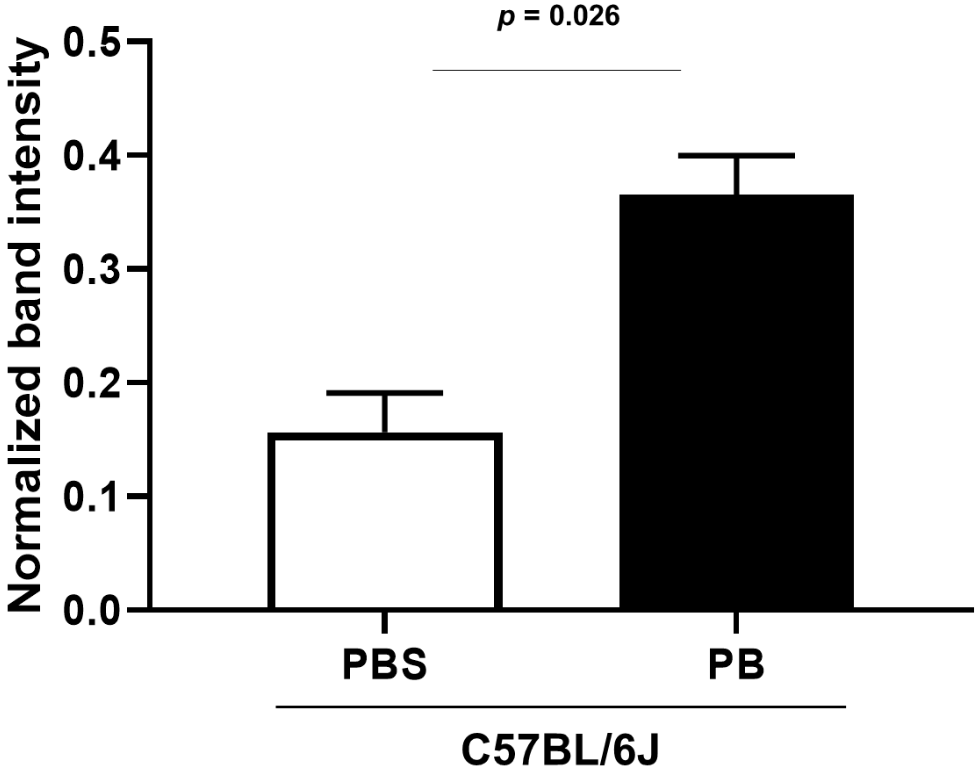

64

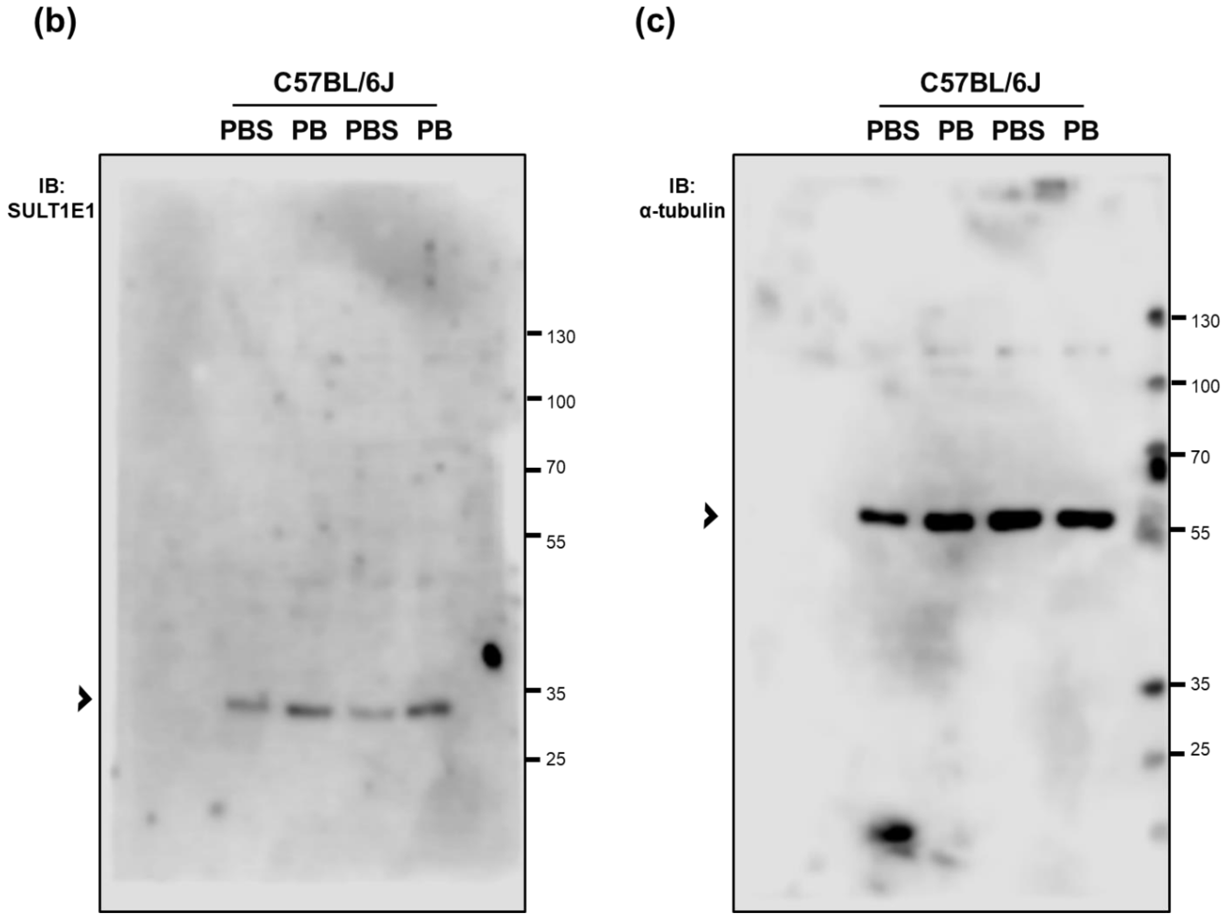
(b)
C57BL/6J
PBS PB PBS PB
IB: SULT1E1
130
100
70
55
35
25
(c)
C57BL/6J
PBS PB PBS PB
IB: α-tubulin
130
100
70
55
35
25

Supplement figure 2.

(a)

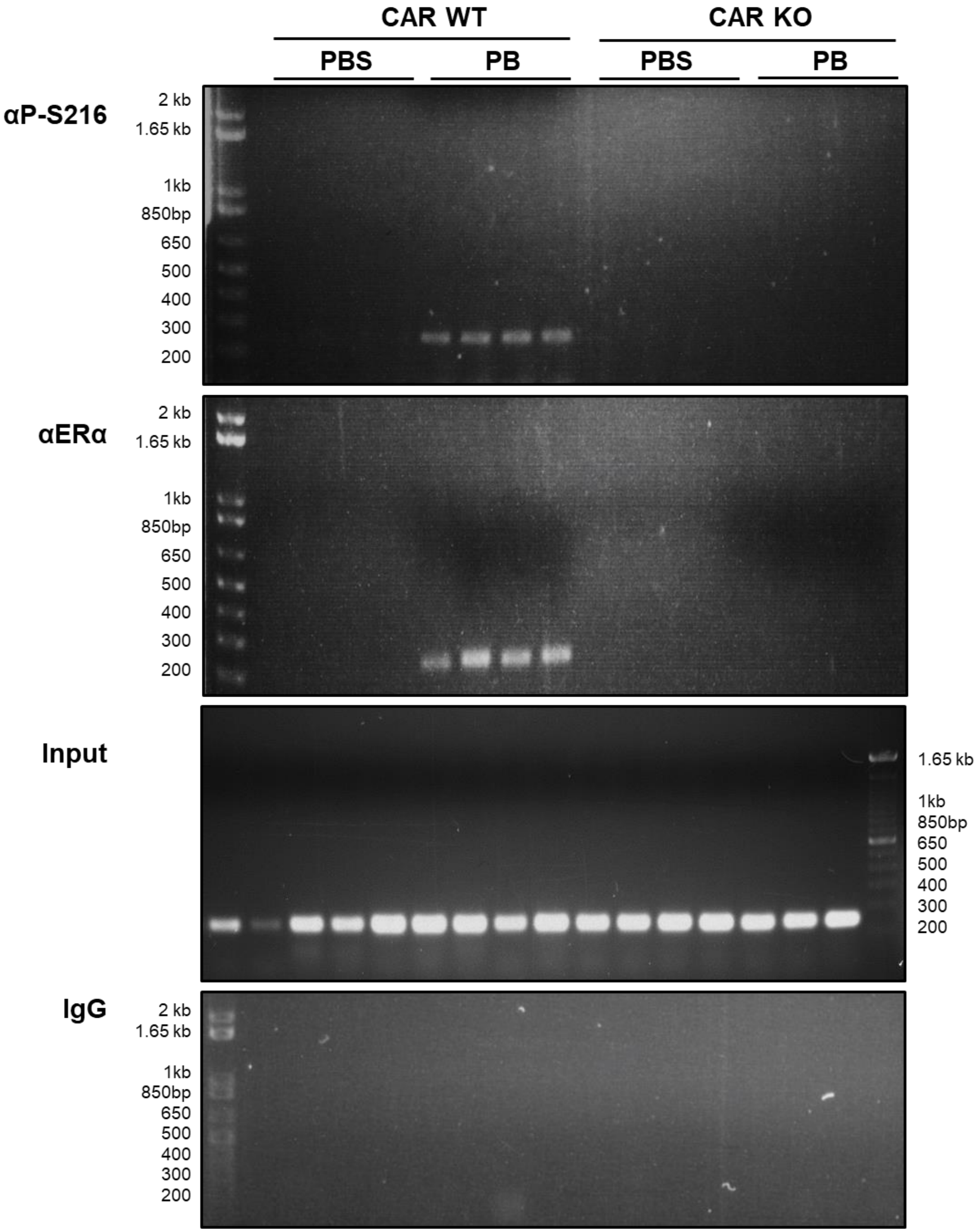

66

(b)

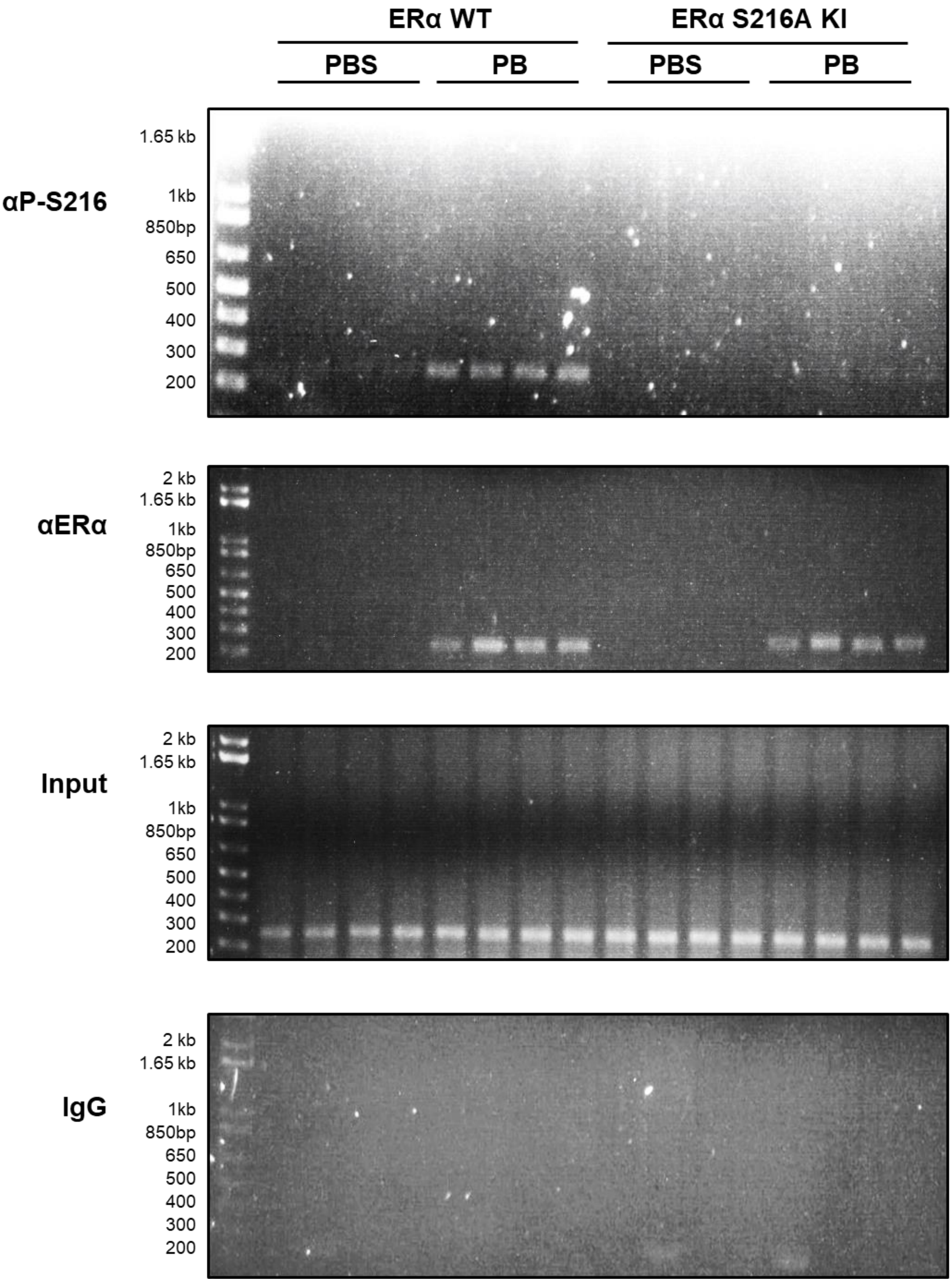
ERα WT
ERα S216A KI
PBS
PB
PBS
PB
αP-S216
1.65 kb
1kb
850bp
650
500
400
300
200
αERα
2 kb
1.65 kb
1kb
850bp
650
500
400
300
200
Input
2 kb
1.65 kb
1kb
850bp
650
500
400
300
200
IgG
2 kb
1.65 kb
1kb
850bp
650
500
400
300
200

67

Supplement figure 3.

(a)

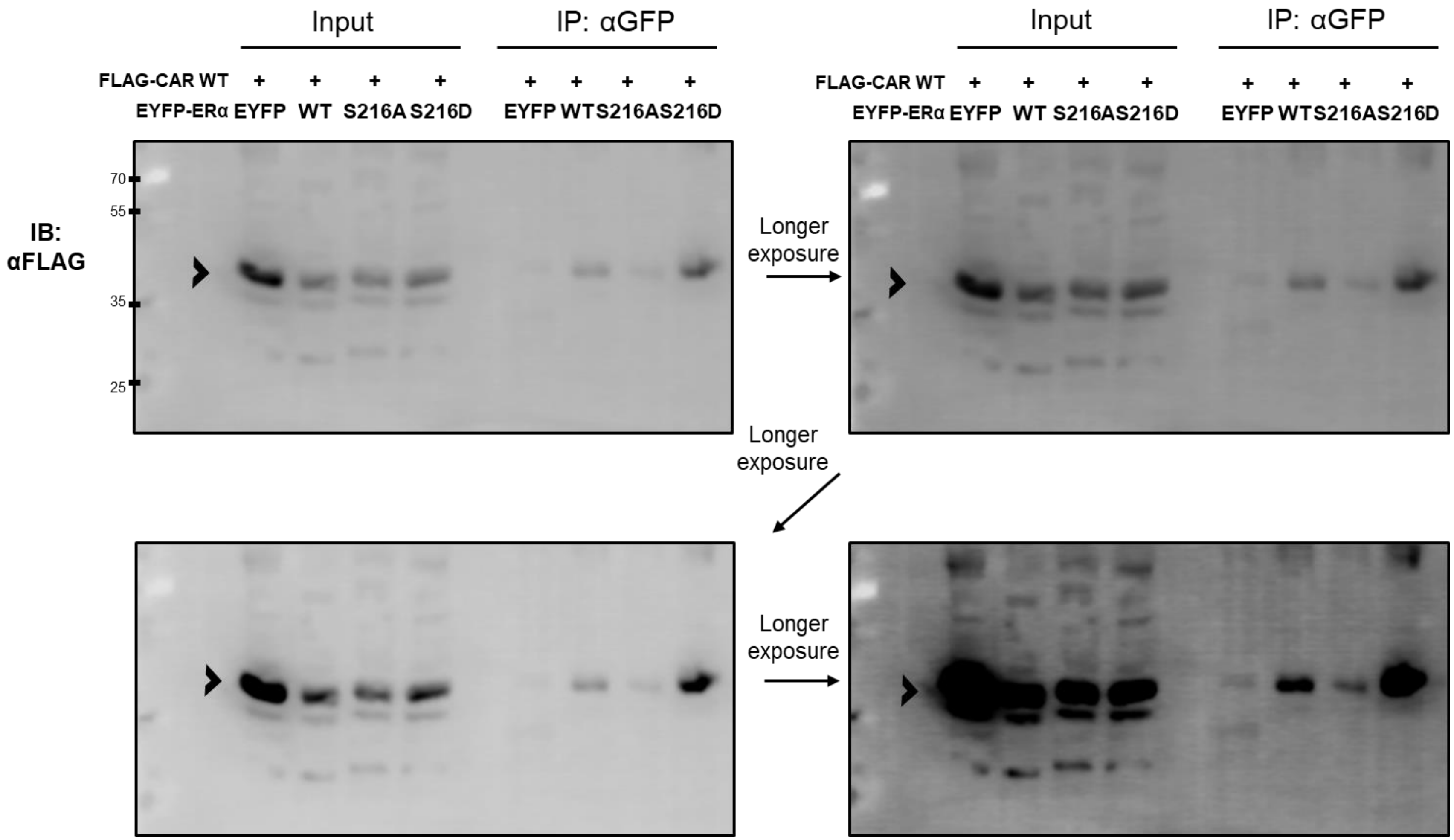

1

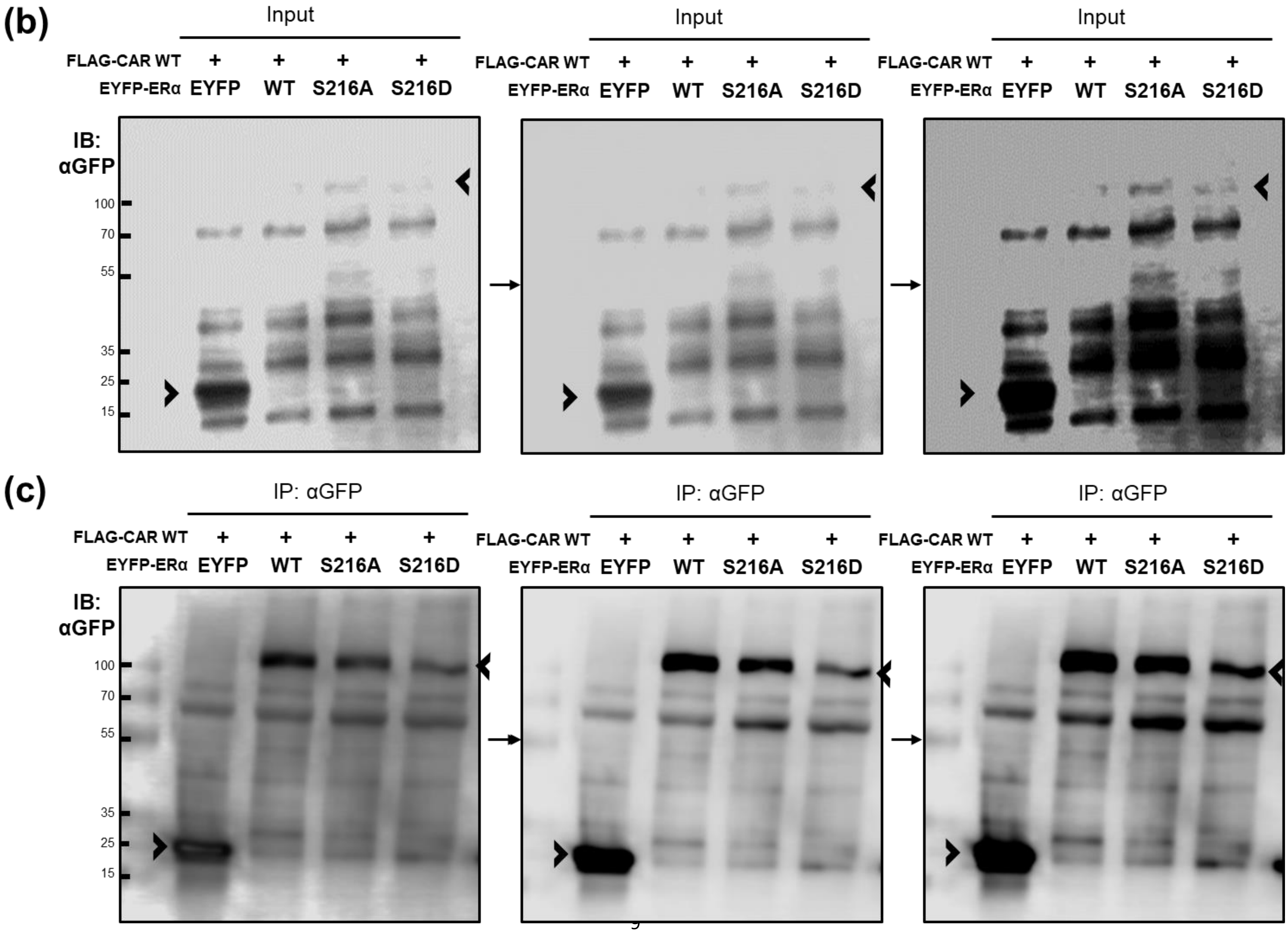
(b)
Input
FLAG-CAR WT + + + +
EYFP-ERα EYFP WT S216A S216D
IB: αGFP
100
70
55
35
25
15
(c)
IP: αGFP
FLAG-CAR WT + + + +
EYFP-ERα EYFP WT S216A S216D
IB: αGFP
100
70
55
35
25
15

Supplement figure 4.

(a)

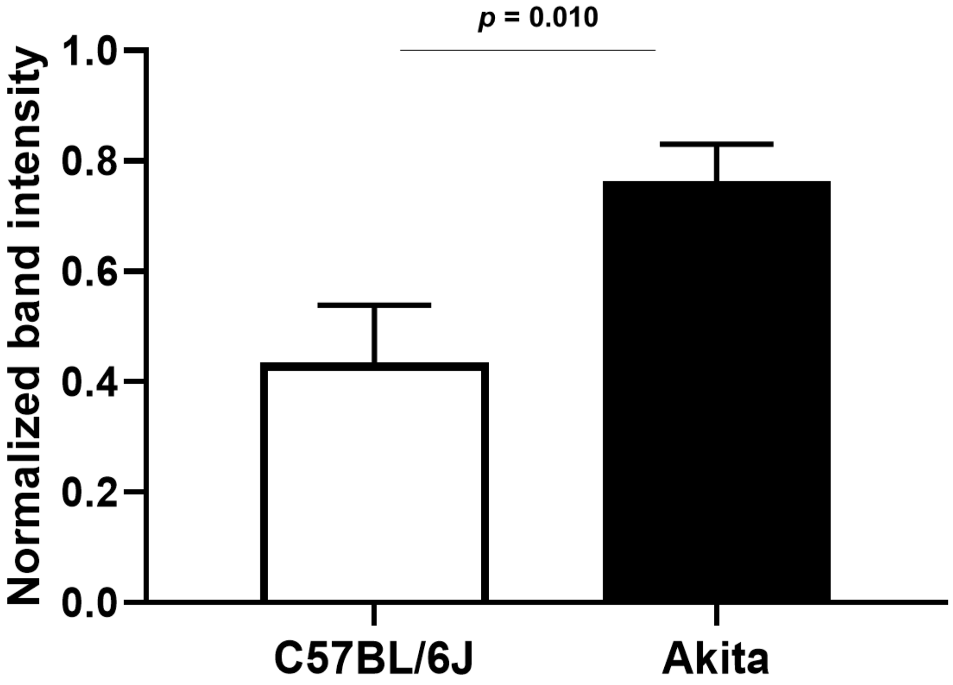

(b)

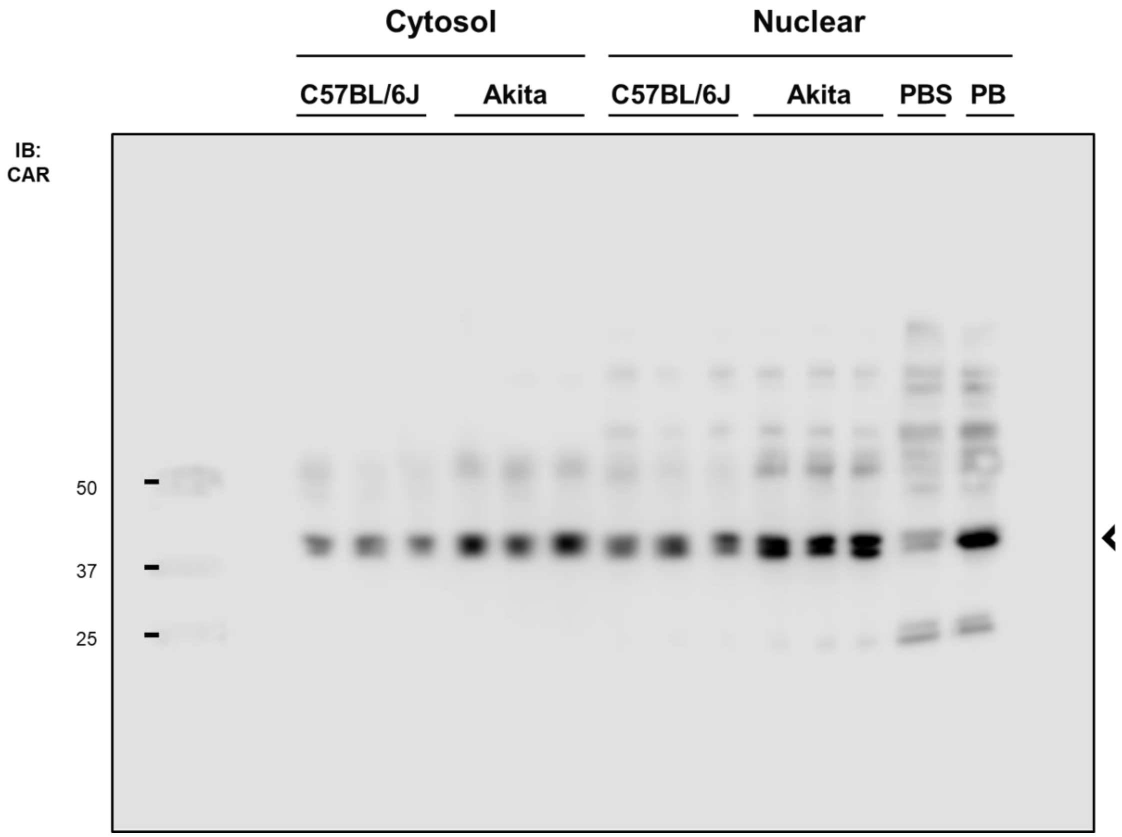
Cytosol
Nuclear
C57BL/6J
Akita
C57BL/6J
Akita
PBS
PB
IB:
CAR
50
37
25

2

(c)

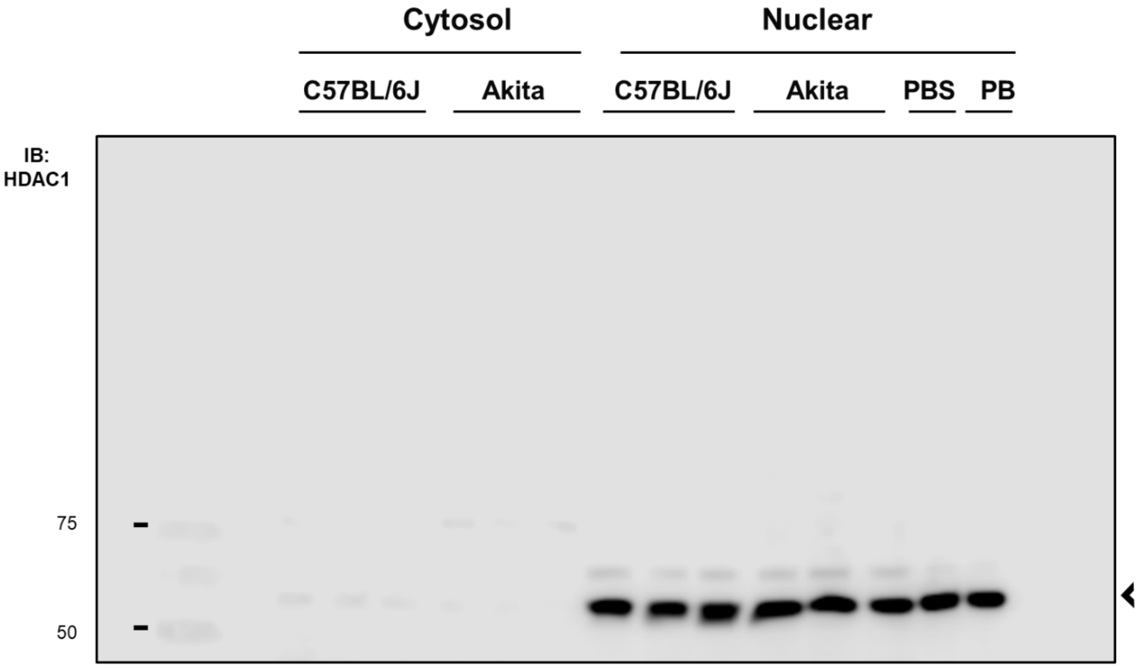
Cytosol
Nuclear
C57BL/6J
Akita
C57BL/6J
Akita
PBS
PB
IB:
HDAC1
75
50

3

Supplement figure 5.

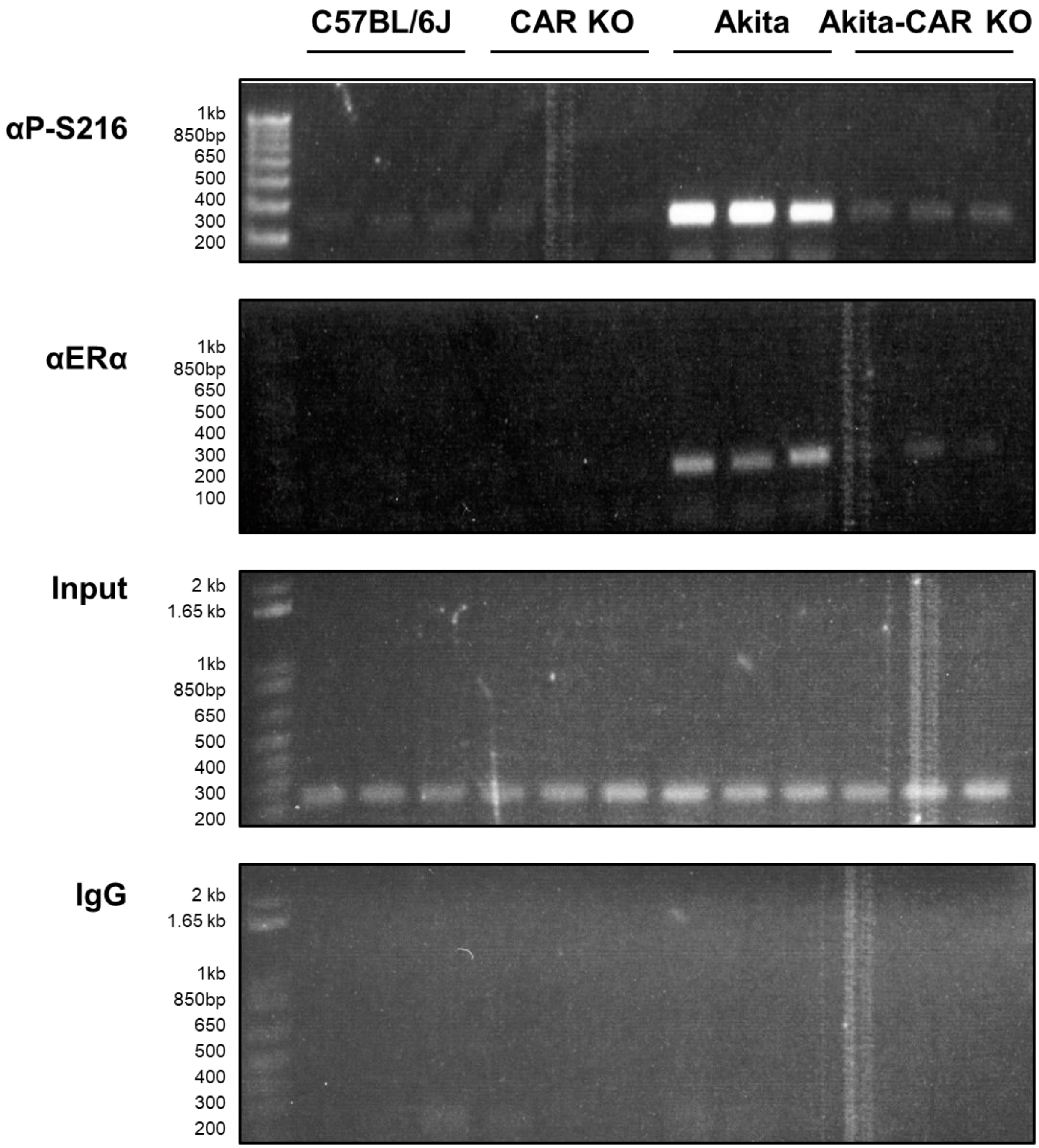

4

Supplement figure 6.

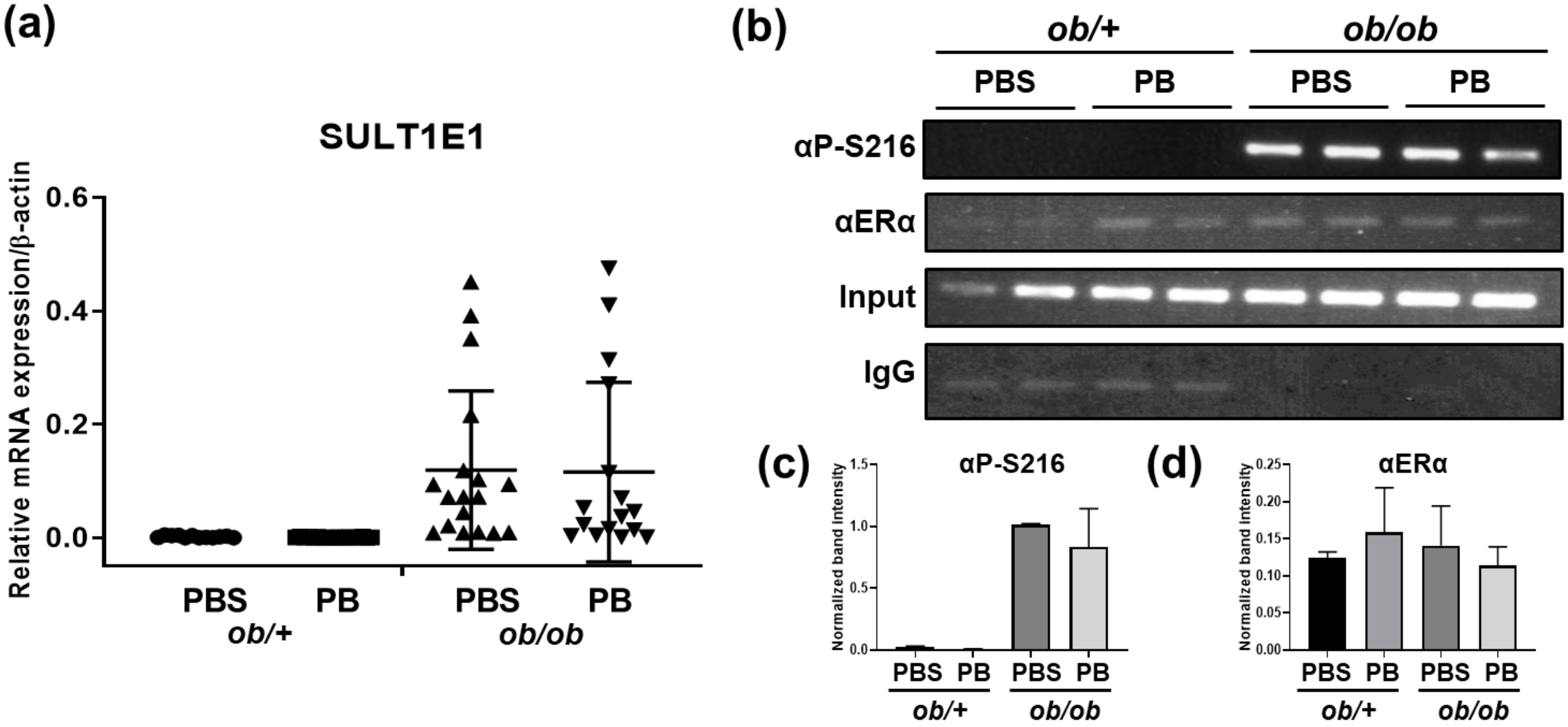

1

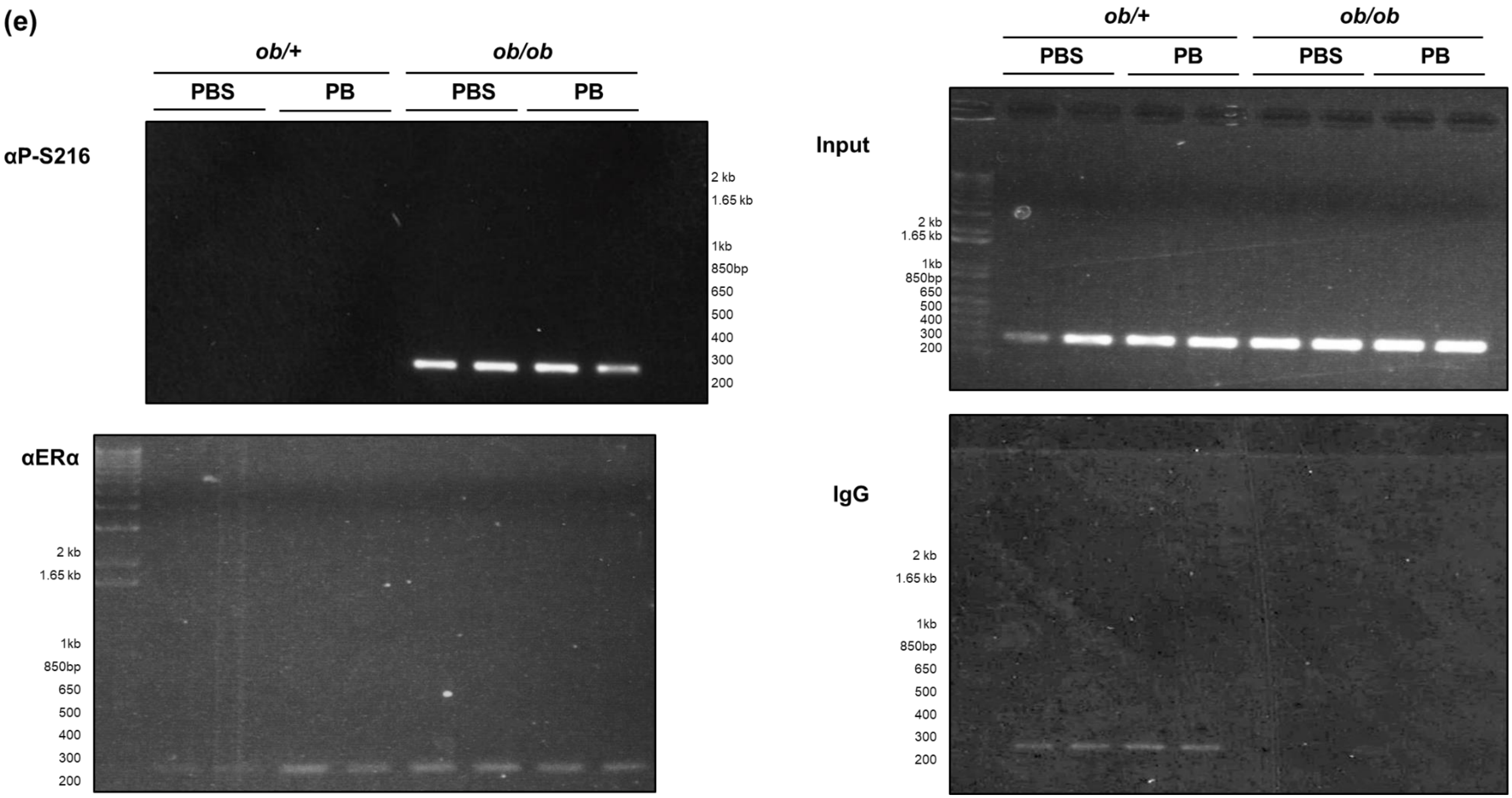
(e)
ob/+
ob/ob
PBS
PB
PBS
PB
αP-S216
2 kb
1.65 kb
1kb
850bp
650
500
400
300
200
αERα
Input
IgG

Supplement figure 7.

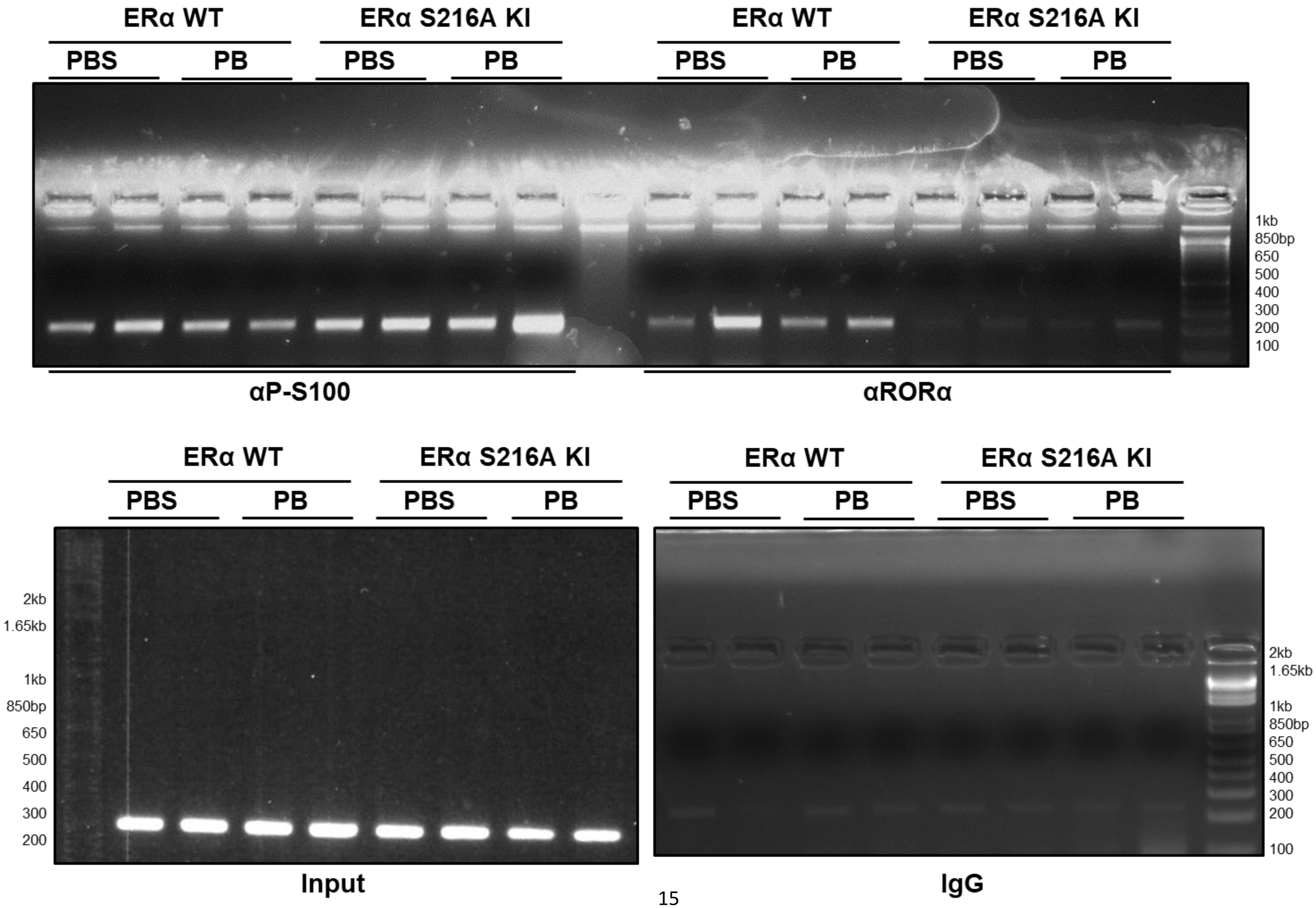

Supplement figure 8.

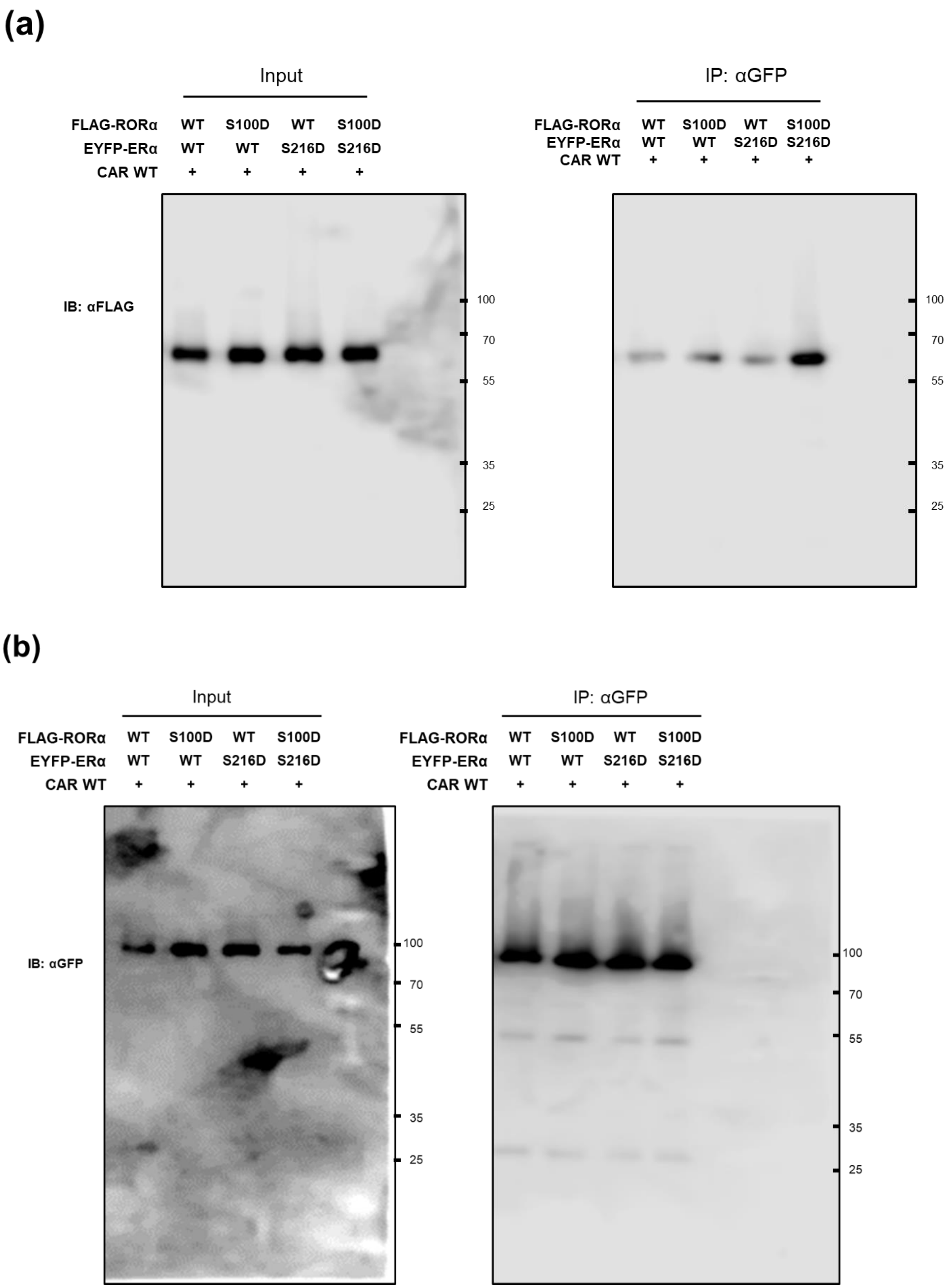

Supplement: Supplementary file 1 — Supplementary information. [file 41598_2020_61767_MOESM1_ESM.pdf]
